# Automatic B cell lymphoma detection using flow cytometry data

**DOI:** 10.1186/1471-2164-14-S7-S1

**Published:** 2013-11-05

**Authors:** Ming-Chih Shih, Shou-Hsuan Stephen Huang, Rachel Donohue, Chung-Che Chang, Youli Zu

**Affiliations:** 1 Department of Computer Science, University of Houston, Houston, Texas, USA; 2 Department of Pathology and Genomic Medicine, The Methodist Hospital, Houston, Texas, USA; 3 Department of Pathology, University of Central Florida, Orlando, Florida, USA

## Abstract

**Background:**

Flow cytometry has been widely used for the diagnosis of various hematopoietic diseases. Although there have been advances in the number of biomarkers that can be analyzed simultaneously and technologies that enable fast performance, the diagnostic data are still interpreted by a manual gating strategy. The process is labor-intensive, time-consuming, and subject to human error.

**Results:**

We used 80 sets of flow cytometry data from 44 healthy donors, 21 patients with chronic lymphocytic leukemia (CLL), and 15 patients with follicular lymphoma (FL). Approximately 15% of data from each group were used to build the profiles. Our approach was able to successfully identify 36/37 healthy donor cases, 18/18 CLL cases, and 12/13 FL cases.

**Conclusions:**

This proof-of-concept study demonstrated that an automated diagnosis of CLL and FL can be obtained by examining the cell capture rates of a test case using the computational method based on the multi-profile detection algorithm. The testing phase of our system is efficient and can facilitate diagnosis of B-lymphocyte neoplasms.

## Background

Flow cytometry (FC) involves conjugating fluorochromes to antibodies, allowing them to bind different cell biomarkers, and passing the stained cells through the path of a laser where the fluorochromes are excited and fluorescence emission is measured. Forward and side scatter of cells give information about the size and complexity of the cells. FC is a valuable tool in the diagnosis of lymphocytic neoplasms. Most of the current software supplied by the cytometer manufacturer provides a 2-parameter visual representation of the multi-dimensional data. Pathologists must manually select the areas that include the cells of interest and view these cells using two other attributes, a process known as gating. These areas of interest are not fixed due to instrument, operator, and sample differences. The pathologists use the clustering of the cells, the distribution and cell size of a cluster, and the relative location of the clusters to make the selection. The process is tedious, time-consuming, and subject to bias. Thus, there is an urgent need to develop a fast and unbiased diagnostic approach [1].

Our ultimate goal is to establish an automated process for clustering cells of interest to replace manual gating [2,3]. Cell populations can be identified in an automated fashion (automated gating) by employing clustering algorithms. The most challenging aspect of the automated process is finding the best clustering algorithm for high-dimensional data sets [4-7]. Many existing dimension-reduction approaches may cause useful information to be lost [8-13]. There have been several attempts to use machine learning technique to automate the gating process [14-20]. The most commonly used approach is the k-mean algorithm [21], which assigns a cell to its nearest cluster. There are several versions of k-mean algorithms such as fuzzy k-mean, K-medoid, Gath Geva, and the Gustafson Kessel algorithm [22]. Other common approaches are hierarchical clustering [23-26] and density-based clustering [27].

Recently, model-based clustering has been gaining popularity [28-31], including use of the expectation-maximization (EM) algorithm [32]. However, most approaches only focus on the first stage of FC data analysis that identifies cell populations, some approaches are only semi-automatic [33], and some only target certain types of lymphocytic neoplasms [34,35]. This paper proposes a novel 3-dimensional (3-D) 5-parameter model that detects multiple types of B-lymphocyte neoplasms.

In this proof-of-concept study, we will apply this methodology to differentiate between selected subtypes of B-lymphocyte neoplasms and identify biomarkers that contribute to the classification of certain subtypes, such as chronic lymphocytic leukemia (CLL) and follicular lymphoma (FL). Our goal is to develop software solutions to allow pathologists to quickly interpret the FC data without bias.

## Methods

### A multi-profile approach for lymphoma detection

The multi-profile lymphoma detection described in this article can detect whether the FC data of an individual matches the profile of a particular type of lymphoma or that of a healthy donor. The objectives of the computational detection system were: (1) minimum human intervention in the detection process, (2) ability to detect various types of B-lymphocyte neoplasms, (3) efficient computation complexity, and (4) reasonable detection rate with a low false-negative rate.

**Figure 1:**
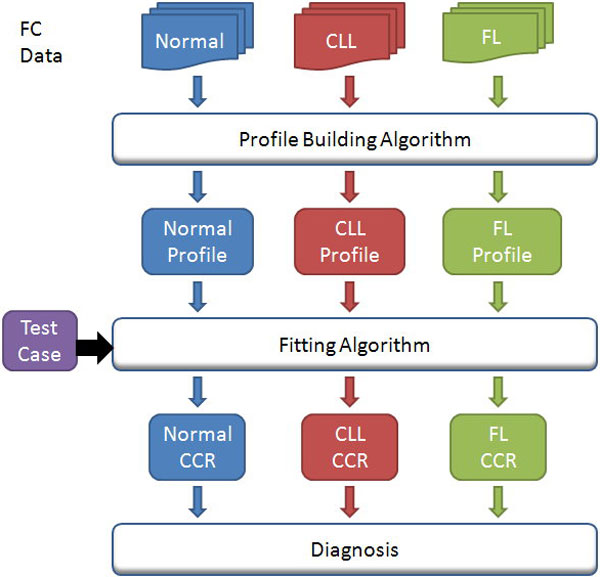
**Overview of the methodology**. Different FC data sets are used to build th*e profiles. Test cases are fitted to the three profiles and the fitting algorithm produces th*ree CCRs. According to the three CCRs, the system gives the diagnosis.

Our system is a multi-anomaly detection system in which a healthy subject's profile and several anomaly profiles are used to determine the closest match. We will first describe a single profile detection system. The system must be trained with known data to gain knowledge about a healthy donor and a patient with a particular type of lymphoma. To achieve the goal of diagnosis, the system has to learn the profile of healthy donors through a training phase and match the profile to the test subject through a fitting (testing) phase. The profile building and fitting will be discussed later. The overall strategy of the testing is summarized in Figure 1. The FC data is categorized into three groups: normal donor, patients with CLL, and patients with FL. A "profile building algorithm" is used to capture the attributes of one individual group, and the profile is a collection of ellipsoids defined on the cytometry map of selected attributes. These ellipsoids are the area of the map where the cells concentrate. One can imagine the profile as a multi-dimensional ellipsoid which filters out some of the fringe outlier cells. A "fitting algorithm" is used to compare a test subject with the profile. Certain features of the profile and the test data have to be extracted for comparison. We needed a metric to measure the fitness of the profile and the test case, defined as the cell capture rate (CCR). This is the ratio of the number of cells captured inside the ellipsoid(s) to that of cells within the test clusters. How the CCR was calculated will be explained in a subsequent section. The CCR estimates how good a test subject matches the profile of healthy donors. Ideally, in a single-profile system, most of the test cells should fall within the ellipsoids defined in the normal profile, thus we can use the CCR to determine if the test case matches the normal profile. In a multi-profile detection system, a test subject then has a vector that includes three CCRs, one each for the normal, CLL, and FL profiles. We will use the vector to diagnose the outcome of the test subject.

### A 3-D 5-parameter flow cytometry data model

**Figure 2:**
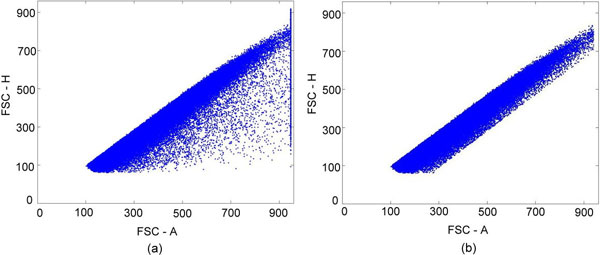
**Removing doublets by forward scatter high (FSC-H) and forward scatter area (FSC-A)**. (a) Before removing doublets and (b) after. The doublets were most likely generated by mechanical error and/or sampling issues.

**Figure 3:**
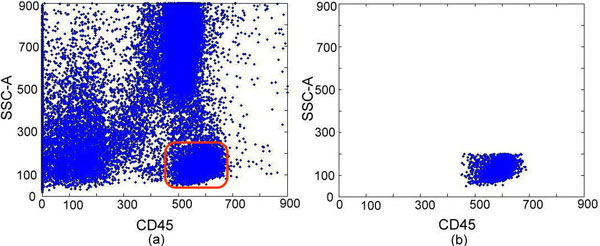
**Gating lymphocytes**. (a) Lymphocytes are gated (circled) by CD45 and SSC-A plots. (b) Selected lymphocytes from (a).

**Figure 4:**
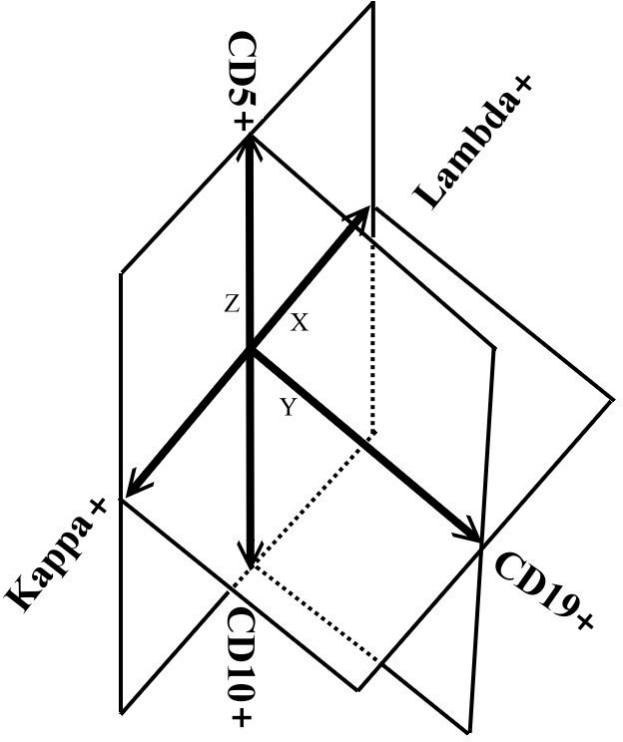
**A novel 3-D 5-parameter model**. The 3-D data view with 5-parameters of CD5, CD10, CD19, Kappa, and Lambda light chains. CD biomarker expression with "+" indicates the difference in expression between two CD biomarkers, e.g., a cell with a CD5+ value means the cell is expressing CD5, and lacking CD10 expression.

Our system focused on characterizing B lymphocyte neoplasms to develop a model-based clustering approach to identify normal or abnormal B cell populations that share similar biological functions. There were two steps in the pre-processing: (1) removal of the doublets (Figure 2) and (2) selecting the lymphocytes (Figure 3). The algorithm for removing the doublets is relatively simple and can be found in [36]. Our system was designed to learn from the user's knowledge and to select and classify these cells automatically. There were five Cluster of Differentiation (CD) biomarkers that were used for data modelling: CD5 (labelled with PerCP), CD10 (APC), CD19 (APC-Cy7), kappa light chain (PE), and lambda light chain (FITC). In addition, CD45-AmCyan was used for lymphocyte gating. Normal B lymphocytes are positive for CD19, but not express (negative for) CD5 and CD10, and composed of nearly even populations of cells expressing kappa or lambda light chains. In CLL, the lymphoma cells are positive for CD19 and CD5, but negative for CD10. In contrast, FL cells are positive for CD19 and CD10, but negative for CD5. Importantly, cells of CLL and FL exhibit kappa or lambda light chain restriction (a feature of malignancy). The five lymphocytic biomarkers were combined into a 3-D image as shown in Figure 4. The x-axis represents kappa light chain and lambda light chain. The y-axis represents CD19, a pan-B cell marker. The z-axis is used for CD5 and CD10 expression. Since a B lymphocyte is either kappa light chain or lambda light chain positive, we subtracted the value of kappa by lambda to eliminate the background signal; likewise, the value of CD5 minus CD10 is presented in the z-axis. We use the notation of "Lambda+" and "Kappa+" to indicate the difference in expression between the two CD biomarkers. A cell with a Lambda+ value in Figure 4 means the cell is expressing Lambda, and lacking Kappa expression. "CD5+" and "CD10+" are used in the figure in a similar fashion. For y-axis, only one biomarker is used, and the cell expressed with CD19 is noted as "CD19+". By analyzing the digital FC data in this novel 3-D 5-parameter model, lymphoma cells can be easily distinguished from the normal cell population and further classified into three sub-types: (a) B cell lymphoma with immunophenotyping prolife CD5+, no CD10-expression, light chain restriction (expressing kappa or lambda alone): this profile could be seen in CLL, small lymphocytic lymphoma (SLL), mantle cell lymphoma (MCL), and CD5+ diffuse large B cell lymphoma (DLBCL), (b) B-cell lymphoma with immunophenoting prolife CD10+, no CD5-expression, light chain restriction: this profile is specific for FL, Burkitt lymphoma, and CD10+ DLBCL, and (c) B-cell lymphoma with immunophenotyping prolife no CD5- or CD10-expression, light chain restriction: including MCL, DLBCL, and other non-classified B cell lymphomas. Notably, this model is expandable and can be used to analyze any type of B lymphocyte neoplasm. For proof-of-concept demonstration, the two most common B cell lymphomas, CLL and FL, were studied. We will include additional neoplasms as the data becomes available.

### Profile building

**Figure 5:**
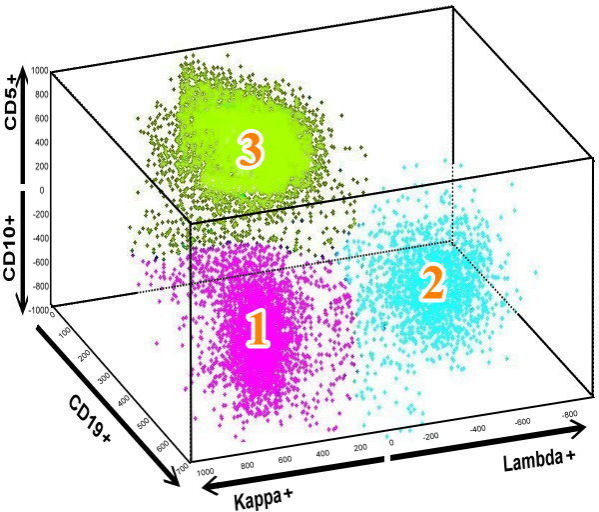
**Lymphocytes in the 3-D 5-parameter data view model**. Lymphocytes from a representative healthy donor. (1) pink cells: B lymphocytes expressing kappa light chain, (2) blue cells: B lymphocytes expressing lambda light chain, and (3) green cells: T lymphocytes lacking CD19 expression.

After removing the doublets and selecting the lymphocytes by gating on CD45 and side scatter, the healthy donors' FC data was plotted using the 3-D 5-parameter model defined above (see Figure 5). Our objective was to identify all clusters so that the lymphocytes can be characterized by the clusters in the 3-D 5-parameter model. In Figure 5, a healthy donor's cells form three clusters: (1) pink cells: B lymphocytes expressing kappa light chain, (2) blue cells: B lymphocytes expressing lambda light chain, and (3) green cells: T lymphocytes.

**Figure 6:**
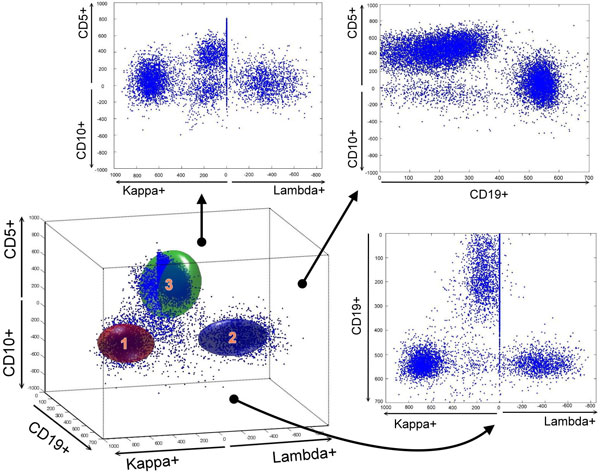
**Lymphocytes in 3-D 5-parameter data view model**. This is an example of using the normal profile to test a healthy donor. The normal profile is represented as three clusters. The corresponding 2-D projections are also provided for review.

The EM algorithm was used to cluster the cells because it gave better results in most of our cases. We used the EM algorithm to produce the final clustering result with means and covariances, and an ellipsoid was constructed with the means and covariances of clusters. The means gave us the center of the ellipsoid and the covariances gave us the orientation and the shape of the ellipsoid. The standard deviation dictated the size of the ellipsoid. Once the means, covariances, and standard deviation were determined, the three ellipsoids for healthy donors were constructed (Figure 6). In other words, the orientation, size, and location of the ellipsoid were considered as the profile of this cluster of healthy donors.

The details of the algorithm are listed below.

Input:

*n*: number of observations

*d*: number of attributes in the observation (3 in the 3-D 5-P model)

*k*: number of clusters (*k = 3 *for normal profile and *k = 2 *for patient profile)

*X*[*i, j*]: observation data of size *n *× *d, i = 1, ..., n *and *j = 1, ..., 3*.

*m*: multiplier for the standard deviation used to determine the size of the ellipsoids (*m *= 2 in our analysis)

Output: *k *ellipsoids containing data points within *m *× std of the centers of the clusters represented by:

*W*[*c*]: percentage of the data points in cluster *c, c = 1, ..., k*.

*M*[*c, i*]: the *i*-th attribute of the of the *c*-th mean of the cluster (*k *× *d*)

*V*[*c, i, j*]: the co-variance matrix of the *c*-th cluster (*k *× *d *× *d*)

Step 1: [Initialization] Given X, use the K-mean algorithm to find *k *clusters of X. The output of K-mean are: *M^(i)^*, *V^(i) ^*and *W^(i)^*, the means, co-variance, and the weight of the k clusters.

Step 2: [Clustering] Use the EM (expectation maximization) algorithm to compute a better clustering of × with initial values *M *= *M^(i)^*, *V *= *V^(i)^*, and *W *= *W^(i)^*.

Step 3: [Ellipsoid Construction] Construct *k *ellipsoids with Means *M *and Co-variance *V *and weight *W*. The ellipsoid should include all data points within *m *× std of the center of its cluster.

**Figure 7:**
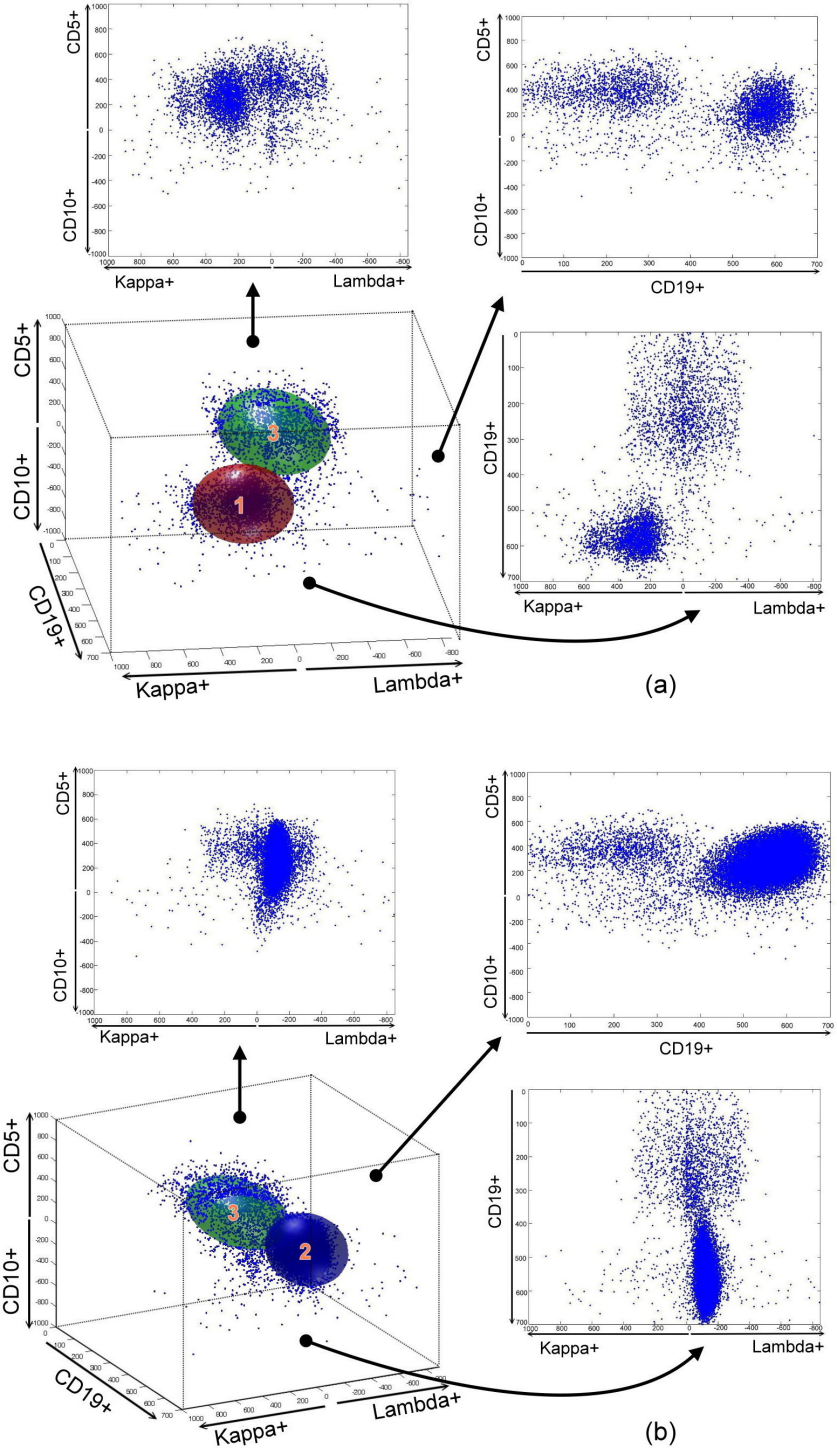
**CLL profile**. The CLL profile is represented as two clusters: kappa chain-restricted (a) and lambda chain-restricted (b).

The process of building a cancer patient's profile (ellipsoids) was the same as that for the healthy donors except the training data were selected from patient cases. However, the modelling of the patient's profile was more complicated. Although most healthy donors show very similar representation in the 3-D 5-parameter model, CLL data are more diverse, most likely due to different stages and severity of disease. While healthy donors have two cell clusters for kappa and lambda light chains, CLL patients show only one cluster because lymphoma cells are restricted to either kappa or lambda light chains. Thus, in the profile-building algorithm, there are only 2 clusters (k = 2) instead of 3 clusters. For example, in Figure 7a, CLL patient 453 only showed one cluster of cells expressing kappa light chain, which is defined as kappa dominant; in Figure 7b, CLL patient 338 showed B cells that were lambda light chain dominant. It is very important to have a good CLL profile to test the subjects, and this can be cross validated by subsequent experiments.

**Figure 8:**
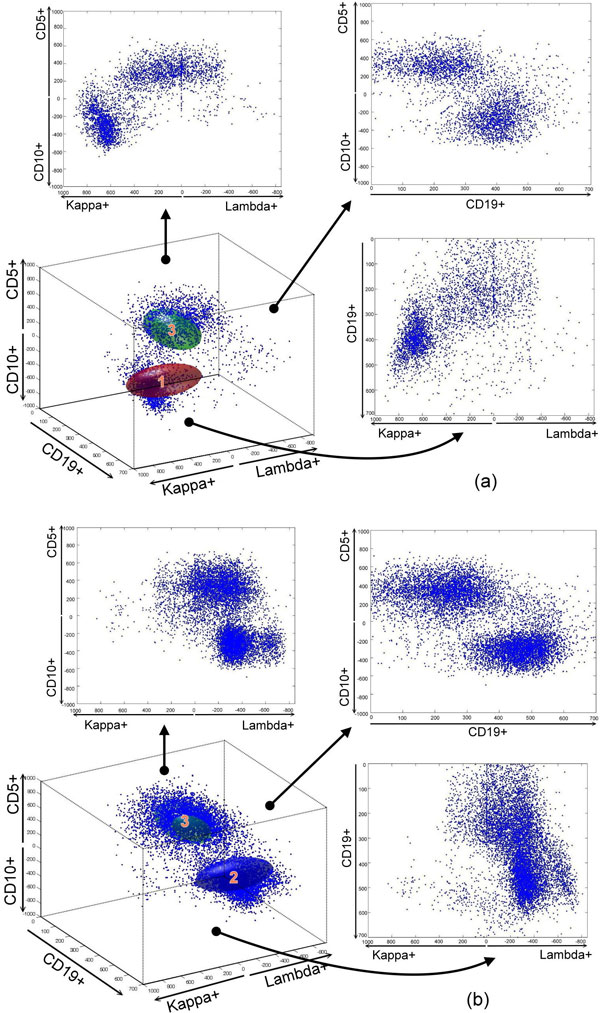
**FL profile**. The FL profile is represented as two clusters: kappa chain-restricted (a) and lambda chain-restricted (b).

The FL profile was built by a similar approach, but both clusters 1 and 2 cells are present in only the CD10+ side of the z-axis (CD5-CD10) since FL cells express only CD10 and lack CD5 expression. For example, in Figure 8a, FL patient 1444 only showed one cluster in the kappa light chain and is defined as kappa dominant. In Figure 8b, FL patient 1284 showed B lymphocytes that were lambda light chain dominant.

### Profile-fitting algorithm

**Figure 9:**
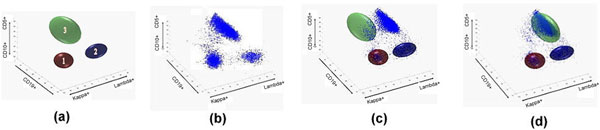
**Testing process**. The process of using the healthy profile to test a normal subject with shifting cells. (a) A normal profile with 3 ellipsoids (b) A normal test case before alignment with the normal profile (c), and after alignment with the normal profile (d).

Once we had constructed the normal profile, a test case (Figure 9b) was "fitted" to the profile (Figure 9a). Our goal was to use these ellipsoids to capture cells of a test case, and count the numbers of captured cells inside an ellipsoid. After the means and co-variances were computed by the normal profile building algorithm, the three profiles were represented as ellipsoids (Figure 9a). However, due to manual handling of the samples, environmental conditions, and the calibration of the instrument, the cell clusters and the ellipsoids may not align very well. An example of cells of a normal test case overlaid with the three-ellipsoid profile is shown in Figure 9c. Thus, a fitting algorithm was required to realign the cell clusters to match with the ellipsoids.

The ratio of the number of cells captured inside the ellipsoid(s) and that of cells of the test clusters is defined as the CCR of the profile on the test case. In other words, the ratio calculation requires two numbers: the number of B cells and the number of overall cells. The number of cells captured by a profile ellipsoid can be used as the numerator of CCR. For the denominator, there are three possibilities: all blood cells, all lymphocytes, or B cells. In the next two paragraphs, we shall describe how the CCR is computed.

To find out the B cells captured by an ellipsoid in the profile, it was necessary to partition the cells into clusters. However, most clustering algorithms are ineffective in dealing with clusters that are very close or intersecting each other. Thus, our first step was to use a hierarchical divisive clustering ("top-down") approach by separating the T cells from the rest of the test cells by using the value of CD19. The parameter *k *is defined as the number of clusters (*k = 3 *for normal profile and *k = 2 *for patient profile) and *X[c,j] *represents the observation data of *c*-th cell. In the first step, T cells were identified and assigned with a label *k*. The next step was to find the center of the T cells. This can be easily achieved by calculating the mean of cells with label *k*. Technical variation, such as different operators, machines, etc., may cause the data to shift. Thus, the third step of alignment is to fix the variation by moving the profile to "fit" the test data. We have tried several methods for alignment. In one approach for fitting the normal profile, we divided B cells into two clusters representing lambda light chain dominant and kappa light chain dominant and obtained the centers of the two clusters. Then we aligned the ellipsoids individually to the corresponding center. This approach fails to detect changes in the distance between clusters. In addition, the clustering algorithm used to separate two clusters that are very closely aligned was not very effective and this may result in misclassifications. In our current work, we adopted a hierarchical approach: we first found the center of the T cells in the test case, and then calculated the difference between center of T cells in the profile and test case. Finally, we aligned all ellipsoids by the difference. In our system, we used only the one or two ellipsoids that represent B cells and left out the ellipsoid that represents T cells since we are detecting B lymphocyte neoplasms. After aligning the ellipsoids to the center of the corresponding clusters, we obtained the numbers of the captured B cells, which is the numerator of the CCR.

For the denominator of the CCR, we tried out all the three possibilities mentioned above. If we use the total number of the blood cells as the denominator, the CCR is compressed to a small range thus it is difficult to distinguish healthy donors and patients. In a preliminary paper we reported [37], the B cell CCR is calculated by the number of B cells inside the ellipsoid divided by the total number of lymphocytes.

That approach gave us a higher CCR to compare since the denominator is smaller. Even though the CCR in [37] was able to distinguish the patients from healthy donors, the CCR for healthy donor using the normal profile is somewhat small (about 13% on average). In this paper, we decide to use a third approach by using the total number of B cells as the denominator. This approach gives us a much higher CCR for healthy donor compared with the normal profile (over 80% on average).

The detail of the fitting process is given below, and the final B cell CCR is defined as the ratio of the number of B cells inside the ellipsoid over the total number of B cells. Input:

*n*: total number of observations

*k*: number of clusters (*k = 3 *for normal profile and *k = 2 *for patient profile)

*d*: number of attributes in the observation (*d *= 3)

*X[c, j]*: observation data of the *c*-th cell, *c *= 1, .., *n*, and *j = 1, ..., d*.

*P*: a Profile (Normal, CLL, or FL) including *M*[*c,j*] and *V[c,i,j], c = 1, ..., k*.

Output: Cell capture rate of × against the profile *P*.

Algorithm:

Step 1: [Clustering of cells] This is achieved by a hierarchical divisive clustering approach to identify the T-cells with the CD19 first. Let cluster[*c*] be the cluster of cell c, thus cluster[*c*]=*k *for all cell *c *in the T-cell cluster. For the rest of the cells, use the K-mean algorithm on X[c,j] to find the remaining *k-1 *cluster(s). The B-cell clusters are numbered as cluster *1*, .. , *k-1*.

Step 2: [Finding the centers] For each cluster, find the center MC[c, i] (*c *= 1, ..., *k*, *i *= 1, ..., *d*) of the cluster by computing the mean of the cells in that cluster *i*.

Step 3: [Alignment] Find the difference δ[*k,i*] of T[*k,i*], the centers of the T-cell clusters and M[*k,i*] the centers of the T-cells of the profile P. Modify the means so that the T-cell cluster aligns with the T-cell ellipsoid, i. e., M[*c,i*] = M[*c,i*]+ δ[*k,i*].

Step 4: [Calculating CCR] Computing the cell capture rate (CCR) of B-cell as:

$$CCR=\frac{\sum_{c=1}^{k-1}\left(number\;of\;cells\;in\;the\;c^{th}\;ellipsoid\right)}{\sum_{c=1}^{k-1}\left(number\;of\;cells\;in\;the\;c^{th}clust\;er\right)}$$

### Diagnosis

More formally, we defined the diagnosis decision process by finding the distance of the CCR vector to the axes. The algorithm is described in the equations below. Let D = {Normal, CLL, FL} be the set of all diagnoses, and CCR_j_ be the CCR for each j in D. Compute the distance from the CCR vector of a test case to the corresponding axis as

$$d_{i}=\sqrt{\underset{j\;\neq \;i}{\sum }CCR_{j}^{2}},\;$$

and

$$\text{Diagnosis}=arg\;\underset{i\in D}{\text{min}}{\;d}_{i}.$$

The algorithm calculates and selects the axis with the smallest distance to the CCR of the test case.

## Results

### Single-profile testing

**Figure 10:**
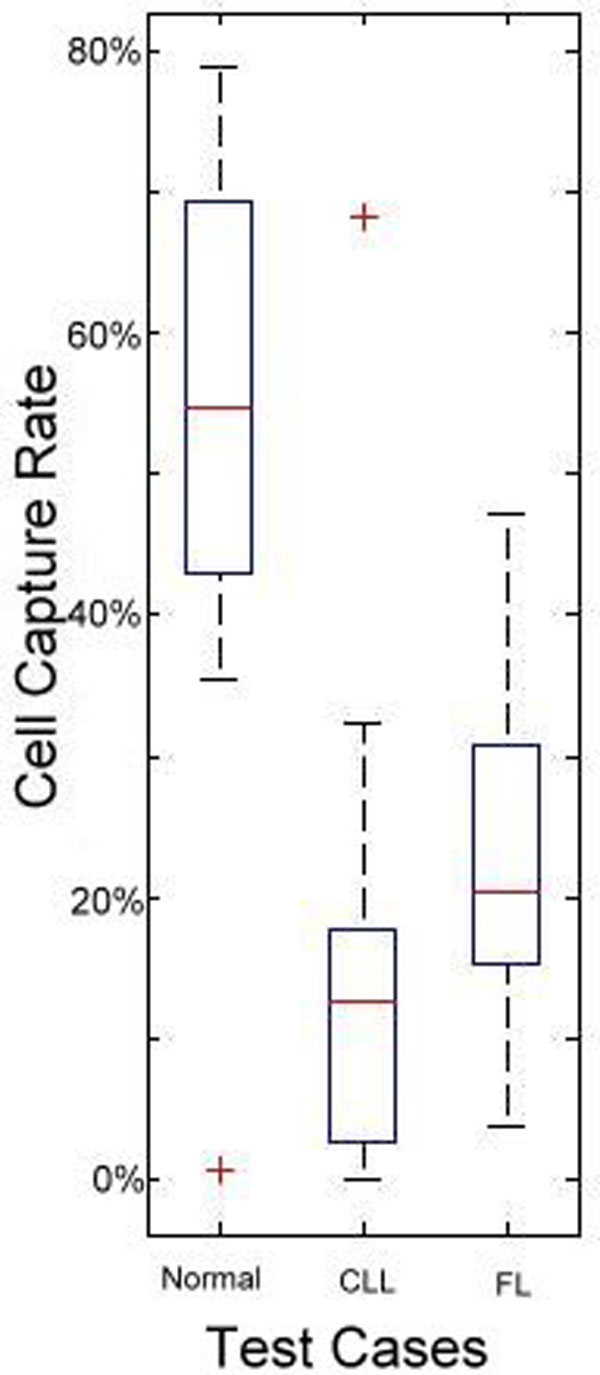
**Single profile testing**. The box plot of 60 cases (24 healthy donors, 21 CLL patient cases, and 15 FL patient cases) fitted against the normal profile is shown. The central mark is the median, the edges of the box are 25th and 75th percentiles, and the whiskers extend to the most extreme data points. The outlier subjects are marked as red "+". The default maximum whisker length is 1.5. Points are drawn as outliers if they are larger than q3 + 1.5 × (q3 - q1) or smaller than q1 - 1.5 × (q3 - q1), where q1 and q3 are the 25th and 75th percentiles, respectively. In this figure, CLL and FL data give lower CCR rates since they do not fit the normal profile well.

Single-profile testing means testing against only the normal healthy profile. We used 72 data sets from 36 healthy donors, 21 CLL patients, and 15 FL patients in our experiment. We constructed the normal profile with 12 randomly selected healthy donors and tested it with the remaining 60 cases. Our hypothesis was that the CCR computed in the fitting algorithm of the healthy cases would fit the normal model better than the patient cases. The result is shown in Figure 10. By comparing the CCRs, the CLL cases were distinguishable from the healthy subjects, but there was overlap between the healthy donors and FL patients. Thus, we were not able to identify all patient samples using only the normal profile. To improve these results, we used multi-profile testing, which combines the results of testing against all three profiles (normal, CLL, and FL).

### Multi-profile testing with cross validation

**Figure 11:**
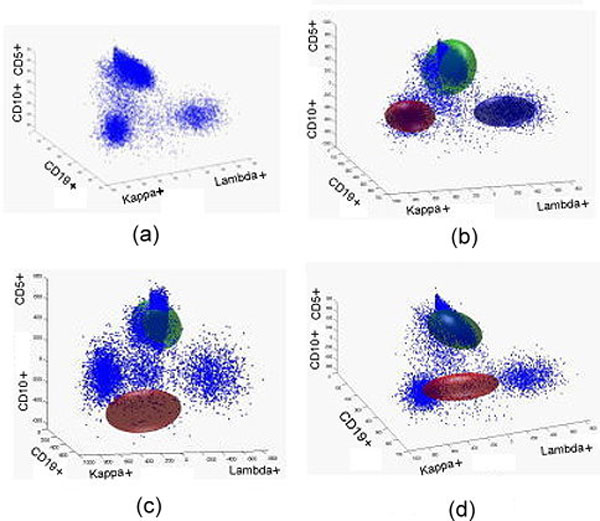
**A test case (healthy) fitted by three profiles**. Figure (a) is a healthy test case. Three profiles, normal, CLL, and FL were fit to the healthy test case. The healthy sample (b) shows alignment, while the CLL and FL profiles do not align (c and d, respectively).

The hypothesis to be tested was that the healthy donors' cases would capture more cells using the healthy subject model, and patients' cases would capture more cells using the patient model. In Figure 11 we show a test case (healthy subject) against all three profiles, and as it is a healthy subject, the normal profile fits better.

The unresolved issue is how to select a suitable profile to best represent the healthy donors, and CLL and FL patients. Building normal profiles is easier since samples from healthy donors are generally consistent and less variable; building patient profiles is more difficult since patient samples can vary dramatically. As a first step we used cross validation to test our approach. Because of the differences noted previously, we used a 3-fold cross validation for building and fitting the normal profile, and leave-one-out cross validation for building and fitting the disease profile. We did not use the leave-one-out method for healthy profile testing for two reasons: (1) leave-one-out will create a lot more cases, and (2) healthy donor samples are more homogenous than CLL and FL cases. Thus we use a 3-fold validation technique for the testing against the normal profile. In this experiment, we used the same 72 data sets from 36 healthy donors, 21 CLL patients, and 15 FL patients (summarized in Table 1). The normal profile is built by merging 12 healthy donors (3-fold) and processed by our profile-building algorithm. Then 60 CCR_normal_ (from testing the 24 remaining healthy donors, 21 CLL patients, and 15 FL patients) are obtained by processing our profile-fitting algorithm. Since it is 3-fold cross validation, we then have 180 CCRnormal (72 normal, 63 CLL, and 45 FL). The CLL profile is built by every CLL subject (leave-one-out) and processed by our profile-building algorithm. Then 72 CCR_CLL_ (from testing 36 healthy donors, 20 remaining CLL patients, and 15 FL patients) obtained by processing our profile-fitting algorithm. Since it is leave-one-out cross validation, we then have 1491 CCR_CLL_ (756 normal, 420 CLL, and 315 FL). Doing similar processes for FL patients, we then have 1065 CCRFL (540 normal, 315 CLL, and 210 FL). Averaging the CCR produces the results shown in Table 2. The average of the 108 CCR_normal_ is 69.9%, which means 69.9% of B cells are inside the normal ellipsoid/profile for healthy donors. In other words, 69.9% of the B cells can be captured by our normal profile. The CLL profile has a 39.5% capture rate for CLL cases, and the FL profile has a 65.7% capture for FL cases. For each test case, we obtained three cell capture rates (CCR_Normal_, CCR_CLL_, and CCR_FL_). By applying the diagnosis decision formula, our system decides which profiles fit better. Based on the cross validation, there are 54,810 test cases and the result is shown in Table 3. Our system can identify 80.7% of healthy donors from all the healthy donors, 61.1% of CLL patients among all the CLL patients, and 64.5% of FL patients among all the FL patients.

**Table 1:** Cross validation.

Profile			
	
	Normal 3-fold	CLL Leave-one-out	FL Leave-one-out
Total Cases	36	21	15
Training	12	1	1
Testing	(24+21+15)x3 = 180	(36+20+15)x21 = 1491	(36+21+14)x15 = 1065

The table shows case information. By using cross validation, there are 12 cases (3-fold) trained to build the normal profile, and 1 case (leave-one-out) trained to build the patient profile.

**Table 2:** Average CCR.

		Profile		
Average CCR				
		Normal 12 Cases	CLL 1 Case	FL 1 Case
	Normal 1368 events	**69.9%**	9.9%	27.6%
Test	CLL 798 events	22.5%	**39.5%**	14.2%
	FL 570 events	48.5%	7.9%	**65.7%**

The 69.9% is obtained by averaging the 72 healthy donors' CCR_normal_; 9.9% is obtained by averaging the 756 healthy donors' CCR_CLL_; 27.6 % is obtained by averaging the 540 healthy donors' CCR_FL_. From this table, healthy donors fit the normal profile better and test samples fit patient profiles better.

**Table 3:** Accuracy.

		Profile		
Accuracy				
		Normal	CLL	FL
	Normal 22,680 events	**80.7%**	2.2%	16.6%
Test	CLL 18,900 events	23.3%	**61.1%**	15.6%
	FL 13,230 events	32.2%	0.6%	**64.5%**

By applying the diagnosis decision formula, the system decides that each event belongs to one decision (normal, CLL, or FL). The table shows the accuracy. For the FL patients, 8,533 (64.5%) events were diagnosed correctly.

Since we adopted the leave-one-out approach for building for the CLL and FL profiles, some of the cancer patients fit the profile better than others. A more carefully selected profile is needed to improve the accuracy of the diagnosis, which is discussed in the next section.

### Multi-profile testing with a data selection strategy for profile building

As mentioned previously, there is no need to pre-select healthy donors to build the normal profile since healthy donors' samples are fairly consistent in composition. To choose a better ellipsoid to represent the CLL, we used the distance between the center of cluster 3 to 1 (or 2) as our selecting criteria in Figure 7a and 7b. We selected approximately 15% of the CLL cases that have a closer value to the mean of the distance. For FL (Figure 8), we will perform the same process to pre-select 15% of FL data for our training cases. The CLL and FL profiles are built by merging the training cases.

**Figure 12:**
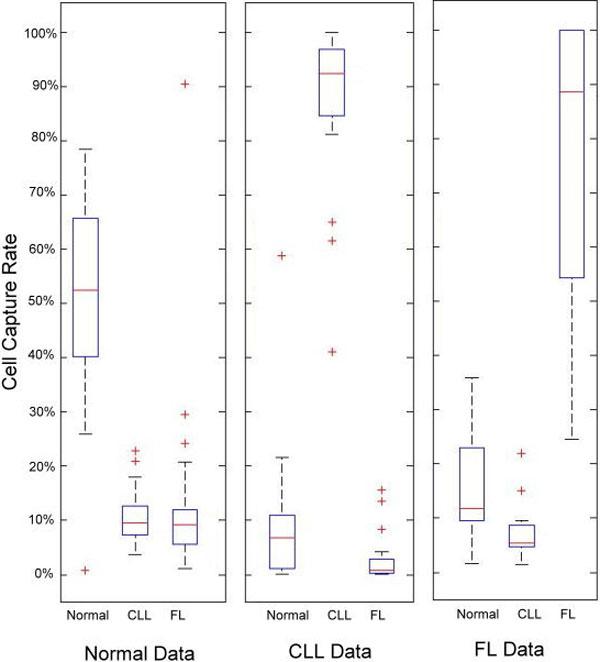
**Multi-profile testing**. The box plot shown represents 60 cases (24 healthy donors, 21 CLL patient cases, and 15 FL patient cases) fitted to the normal, CLL, and FL profiles. The box plot shows the normal data fits the normal profile better, the CLL data fits the CLL profile better, and the FL data fits the FL profile better.

**Figure 13:**
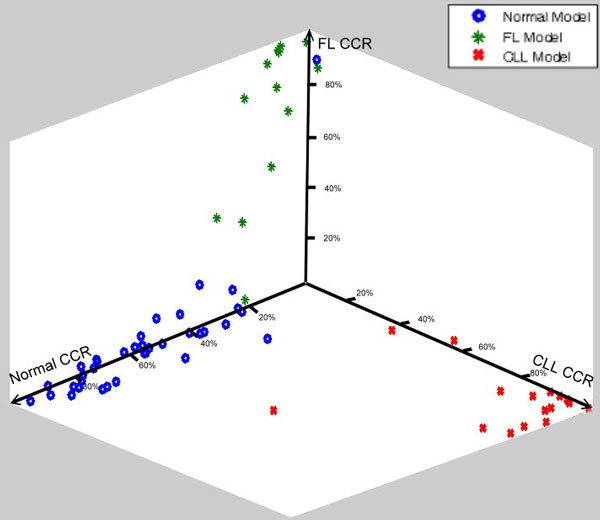
**Result using three profiles**. All 68 data sets were plotted using the three cell capture rates (CCR_Normal_, CCR_CLL_, and CCR_FL_). The majority of test cases aligned to only one axis (only two cases are misclassified).

In this experiment, we used 80 data sets from 44 healthy donors, 21 CLL patients, and 15 FL patients (see Table 4). We used the pre-selected 15% of data to build the profile, and tested the remaining 68 cases. In Figure 12, the average CCR shows a higher value than the average data listed in Table 2. For each test case, we obtained three CCRs. We plotted the test cases using the three CCRs and they clustered in three regions in 3-D space (Figure 13). Because the CCR of the matched profile gave a much higher value than the unmatched ones, most of the test cases were very close to the axis representing the corresponding profile. The final result is shown in Table 5. Our system successfully identified 36 out of 37 normal cases, 18 out of 18 CLL cases, and 12 out of 13 FL cases.

**Table 4:** Multi-profile testing with data selection strategy for profile building.

	Profile		
	
	Normal	CLL	FL
Total Cases	44	21	15
Training	7	3	2
Testing	37	18	13

The table shows the number of cases used to build profiles (7 cases for building normal profile, 3 cases for building CLL profile, and 2 cases for building FL profile). The remaining cases are used for testing purpose.

**Table 5:** Accuracy.

		Profile					
		
Accuracy		Normal 7 Cases		CLL 3 Cases		FL 2 Cases	
		
		Count	%	Count	%	Count	%
	Normal 37 Cases	36	**97.3%**	0	0%	1	2.7%
**Test**	CLL 18 Cases	0	0%	18	**100%**	0	0%
	FL 13 Cases	1	7.7%	0	0%	12	**92.3%**

Our system successfully identified 36/37 normal cases, 18/18 CLL cases, and 12/13 FL cases.

## Conclusions

As a proof-of-concept study, we have demonstrated a multi-profile B lymphocyte neoplasm analysis methodology to automate the detection of certain types of B lymphocyte neoplasms by FC. A profiling method was described that characterized both the healthy donors and patients with different types of B-lymphocyte neoplasms. A CCR was defined to measure the fitness of a test case against the profile. We have demonstrated that one can obtain an automated diagnosis of CLL and FL by examining the CCRs of a test case against all three profiles. Although we only looked at FL and CLL in this study, this novel 3-D 5-parameter detection system should be capable of identifying other types of B lymphocyte neoplasms. Moreover, since the analysis is computational, it is possible to track FC data for monitoring disease progression of a lymphoma patient.

Additionally, this 3-D 5-parameter detection system provides a novel way for pathologists to interpret FC data. Instead of manually gating on numerous 2-parameter plots, they can analyze 5-parameters in a 3-D image that can be rotated and viewed from various angles. This would allow them to see small clusters of cells that may be obscured in a 2-D image. In this way the 3-D 5-parameter detection system has the potential to improve a process that is labor-intensive, time-consuming, and subject to human error through automation and improved data interpretation.

This article is an expanded paper previously presented at the 2012 IEEE 2nd International Conference on Computational Advances in Bio and Medical Sciences (ICCABS) [37]. We expanded the preliminary result presented at the ICCABS conference and added the following new components. 1. Detail algorithms of our method: In the ICCABS paper, we only included the brief descriptions of building profiles and using the profile to test a new subject. In our current submission, we have included the detail steps of the Profile Building Algorithm and Fitting Algorithm. 2. Additional experimental results: After collecting more data from the Methodist Hospital, we added 7 more FL patient cases which almost doubled the FL sample size. 3. A comprehensive analysis including cross-validation of the testing: In the current submission, we added (a) Single-Profile Testing, (b) Multi-Profile Testing with Cross Validation, (c) A data selection strategy for profile building which yields better profiles for CLL and FL. 4: New definition of the B cell CCR: the B cell CCR is calculated by the number of B cells inside the ellipsoid divided by the total number of lymphocytes. 5. Other Improvements: We presented a new overview of the methodology which gives a better explanation of the system, and we used box plots to compare the cell capture rate of using various profiles. This gives reader a better understanding of the distribution of the CCRs.

## Competing interests

The authors declare that they have no competing interests.

## Authors' contributions

SH and CC conceived the idea of a computational model of processing the flow cytometry data. YZ proposed the 3-D 5-parameter data model and draft the manuscript. RD prepared the flow cytometry data collected at the Methodist Hospital under an approved IRB protocol and helped with the manuscript writing. MS and SH conceived and designed the experiment, analyzed the data and wrote the paper. All authors read and approved the final manuscript.

## Declarations

Publication of this article was supported by the Methodist Hospital.

This article has been published as part of *BMC Genomics *Volume 14 Supplement 7, 2013: Selected articles from the Second IEEE International Conference on Computational Advances in Bio and Medical Sciences (ICCABS 2012): Genomics. The full contents of the supplement are available online at http://www.biomedcentral.com/bmcgenomics/supplements/14/S7
